# Role of miR-24-3p and miR-146a-5p in dendritic cells’ maturation process induced by contact sensitizers

**DOI:** 10.1007/s00204-023-03542-z

**Published:** 2023-06-16

**Authors:** Valentina Galbiati, Marine-Alexia Lefevre, Ambra Maddalon, Marc Vocanson, Martina Iulini, Marina Marinovich, Emanuela Corsini

**Affiliations:** 1 Laboratory of Toxicology, DiSFeB, Università degli Studi di Milano, Milan, Italy; 2 CIRI, Centre International de Recherche en Infectiologie, (Team Epidermal Immunity and Allergy), Univ Lyon; Inserm, U1111, Université Claude Bernard Lyon 1; CNRS, UMR5308; ENS de Lyon, Lyon, France

**Keywords:** miRNA, In vitro, Dendritic cells, Allergic contact dermatitis

## Abstract

MiRNAs are non-coding RNA molecules that regulate gene expression at the post-transcriptional level. Although allergic contact dermatitis has been studied extensively, few studies addressed miRNA expression and their role in dendritic cell activation. The main aim of this work was to investigate the role of miRNAs in the underlying mechanism of dendritic cell maturation induced by contact sensitizers of different potency. Experiments were conducted using THP-1-derived immature DCs (iDCs). Contact allergens of different potency were used: p-benzoquinone, Bandrowski’s base, and 2,4-dinitrochlorobenzene as extreme; nickel sulfate hexahydrate, diethyl maleate and 2-mercaptobenzothiazole as moderate; and α-hexyl cinnamaldehyde, eugenol, and imidazolidinyl urea as weak. Selective inhibitor and mimic miRNAs were then used and several cell surface markers was evaluated as targets. Also, patients patch tested with nickel were analyzed to determine miRNAs expression. Results indicate an important role of miR-24-3p and miR-146a-5p in DCs activation. miR-24-3p was up-regulated by extreme and weak contact allergens, while miR-146a-5p was up-regulated by weak and moderate contact allergens and down-regulated only by the extreme ones. Also, the involvement of PKCβ in contact allergen-induced miR-24-3p and miR-146a-5p expression was demonstrated. Furthermore, the expression of the two miRNAs maintains the same trend of expression in both in vitro and in human conditions after nickel exposure. Results obtained suggest the involvement of miR-24 and miR-146a in DCs maturation process in the proposed in vitro model, supported also by human evidences.

## Introduction

Allergic contact dermatitis (ACD) is an undesired side effect in the development of drugs and cosmetic ingredients as well as after contact with environmental or industrial chemicals (Basketter et al. 2008; Nosbaum et al. 2009). Contact sensitizers have been demonstrated to induce phenotypic and functional changes in dendritic cells (DC), enhancing their antigen-presenting capacity that ultimately modulates T-cell response (Morgan et al. 2019).

MicroRNAs are involved in the regulation of gene expression at the post-transcriptional level and they have a major impact on several physiological and pathological cellular processes (Jinnin 2015). The most studied skin pathologies and miRNAs’ implication are psoriasis and atopic dermatitis (Joyce et al. 2011; Schneider 2011). MiRNAs such as miR-21, miR-223, 142-3p and 142-5p appear to be consistently deregulated in different inflammatory skin diseases, including ACD, indicating a common role in biological processes (Vennegaard et al. 2012). These four miRNAs in ACD seems to be related to the increased infiltration and activation of T cells in the skin upon challenge with the allergen (Løvendorf and Skov 2015).

In addition to the hazard identification of skin sensitizers, it is equally important to determine the potency of a sensitizer for a proper risk assessment. Until now, the murine local lymph node assay (LLNA – OECD TG 429) has proved to be very useful in hazard identification but also in assessing the skin sensitization potency of chemicals (Basketter et al. 1996). However, the EU cosmetic regulation 1223/2009 that ban the use of animals for cosmetics ingredients since 2013, has highly promote the development of animal-free methods. With the only exception of the kinetic Direct Peptide Reactivity Assay (kDPRA), a stand-alone assay able to identify GHS 1a sensitizers (TG OECD 442C), the other available validated non-animal test methods are able to identify skin sensitization hazards but not to determine the potency of the chemicals tested (Natsch and Gerberick 2022). The identification of mechanisms influencing the vigor of T-cell responses require a better understanding of the molecular events that trigger cell activation (Corsini et al. 2018).

It is well known that protein kinase C (PKC) plays a key regulatory role in a variety of cellular functions, including cell growth, differentiation, and gene expression. Different PKC isoforms mediate specific cellular signals required for activation, proliferation, differentiation and survival of immune cells (Corsini et al. 2014; Cosentino-Gomes et al. 2012). It has been shown that PKC activation is necessary and sufficient to drive human CD34+ hematopoietic progenitor cells, peripheral blood monocytes or myeloid leukemic cell lines to DC differentiation (Cejas et al. 2005). It has also been reported that one important pathway involved in DC activation is indeed represented by PKCβ activation (Corsini et al. 2014; Galbiati et al. 2020).

The main aim of this study was to investigate the involvement of specific miRNAs in an in vitro DCs’ activation and maturation induced by contact allergens of different potency. After an initial screening and a bibliographic research focused on miRNAs possibly involved in ACD, two miRNAs have been then selected to proceed with further analysis. For this purpose, miR-24-3p and miR-146a-5p have been selected, based on preliminary results obtained and also taking into consideration the lack of information available about their role in ACD. The main goal is to better understand the mechanisms that underlie the potency of chemicals classified as contact sensitizers with an in vitro approach, with additional human evidence.

## Materials and methods

### Chemicals

The following skin sensitizers were used: p-benzoquinone (BZQ), Bandrowski’s base (BB), 2,4-dinitrochlorobenzene (DNCB), nickel sulfate hexahydrate (Nickel), diethyl maleate (DEM), 2-mercaptobenzothiazole (MBT), α-hexyl cinnamaldehyde (HCA), eugenol (EUG) and imidazolidinyl urea (IMZ). The selected contact sensitizers were chosen according to their potency on the basis of the EC3 LLNA values, covering different potencies from extreme to weak (Fig. 2, Panel A). Chemicals were purchased from Sigma Aldrich and Apollo Scientific (Bandrowski’s base) at the highest purity available. BZQ, BB, DNCB, DEM, HCA, EUG and MBT were dissolved in dimethyl sulfoxide (DMSO), while Nickel and IMZ were dissolved in Dulbecco’s Phosphate Buffered Saline (dPBS). Cell culture media and supplements required for cell culture were from Sigma Aldrich.

### Cells

#### DCs’ differentiation

For DCs’ differentiation, THP-1 cells (Elabscience Biotechnology Inc.—Houston, Texas, USA) were treated for 5 days with rhIL-4 (1500 UI/mL—R&D Systems) and rhGM-CSF (1500 UI/mL—R&D Systems) to acquire the properties of immature DCs (iDCs) as described by Berges et al. (Berges et al. 2005). RPMI 1640 containing 2 M glutamine, 0.1 mg/mL streptomycin, 100 UI/mL penicillin, 50 μM 2-mercaptoethanol, supplemented with 10% heated-inactivated fetal calf serum was used and cells were cultured at 37 °C in 5% CO_2_. At Day 5, mature DCs (mDCs) were then generated from iDCs by addition of rhIL-4 (3000 UI/mL), rhGM-CSF (1500 UI/mL), rhTNF-α (2000 UI/mL—Sigma Aldrich) and ionomycin (200 ng/mL—Sigma Aldrich) or by exposure to the selected contact allergen for 24 and 72 h using complete RPMI 1640, without serum.

#### Cell viability

THP-1 cells were treated for 24 h with increasing concentrations of the selected chemicals. Cell viability was assessed by propidium iodide (PI) uptake using flow cytometry with the acquisition channel FL-2 (final concentration of PI 0.5 μg/mL). A total of 10,000 cells were acquired. Vehicle-treated cells (control) were set as 100% and cell viability of allergen-treated cells calculated using the cytometer analysis program. The concentration of allergens resulting in 75% of viability (CV75) was then calculated by linear interpolation using InStat software version 7.0 (GraphPad Software, La Jolla, CA, USA). iDCs were exposed to the same CV75 obtained from the THP-1 evaluation on selected chemicals. Viability of iDCs after treatment was always checked with the same technique described for the THP-1 cells.

#### miRNA mimic and inhibitor conditions

To identify the targets and roles of miR-24-3p and miR-146a-5p, transfection of specific miRNA inhibitor and mimic were performed following Qiagen protocols. Cells with the transfection complexes were incubated for 48 h (until Day 5) and then exposed to BB (2 μg/mL), Nickel (20 μg/mL), HCA (20 μg/mL) or to the maturation cocktail. After 24 and 72 h, cells were analyzed for surface markers expression.

To investigate the role of PKCβ in chemical-induced cell surface markers and miRNAs expression, iDCs were cultured in the presence or absence of a selective cell-permeable inhibitor of PKCβ as previously published (Corsini et al. 2014). In details, iDCs were treated at Day 5 with PKCβ pseudosubstrate (5 μM) for 2 h, and then exposed to BB (2 μg/mL), HCA (20 μg/mL) or to the maturation cocktail for 24 h.

### Patch test procedure and skin samples

Patients suspected for skin allergy were enrolled in the study after being referred to the hospital for routine diagnosis of contact dermatitis. They were patch tested either with the contact allergen nickel. At 72 h, the patch test sites were evaluated for reactivity, and reactions were clinically graded as negative (−), doubtful (+ /?) or positive (1+, 2+ or 3+). Biopsies (3 × 3 mm) were collected from positive patch-tested skin, as well as from vehicle (petrolatum) patch-tested skin. The results of the patch test of the five samples selected for the miRNAs analysis are the following: f4 donors 2+ (P1, P6, P8, and P9) and 1 donor 1+ (P7). The study was approved by Regional Ethical Review Boards (N°19–145 CHU Lyon; Dnr-2017/19 Lund University), and written informed consent was obtained from each participant.

### Cell surface markers’ expression

Cell surface markers were evaluated by flow cytometry analysis. iDCs were treated at Day 5 with BB (2 μg/mL), nickel (20 μg/mL), HCA (20 μg/mL) or to the maturation cocktail in presence or absence of miRNAs mimic and inhibitor. Supernatants were collected for cytokines assessment, and cells stained with specific FITC/PE/Per-CP-conjugated antibodies against CD36, CD40, CD80, CD86, CD206, CCR7 and HLA-DR (BD, Becton Dickinson and ImmunoTools) or with isotype control antibodies at 4 °C following supplier's instructions. The intensity of fluorescence was analyzed using Novocyte3000 flow cytometer, and data were quantified using NovoCyte software (NovoCyte). Changes in surface marker expression are expressed as Stimulation Index (SI) calculated on the Mean or Median Fluorescence Intensity (MFI) values (treated cells/control cells).

### RNA extraction and microRNA reverse-transcription

For RNA extraction, the RNeasy Mini Kit was used (Qiagen), following the manufacturer’s instructions. After RNA extraction and quantification, a specific miRNA reverse-transcription reaction was performed with miScript HiSpec Buffer, contained in the miScript II RT Kit (Qiagen) following the manufacturer’s instructions. 250 ng of RNA was reverse-transcripted for miRNA PCR Array analysis, while 2 μg of RNA was reverse-transcripted for miRNA real-time PCR confirmation.

### microRNA profiling and expression

cDNA prepared in a reverse-transcription reaction was used as template for real-time PCR analysis (ABI 7500—Applied Biosystem) using a miScript SYBR Green Kit (Qiagen). In details, miScript miRNA PCR Array Human miFinder was used (MIHS-001Z), in 96-well format to perform a preliminary microRNA profiling. The consequent miRNAs expression was than confirmed using real-time PCR analysis (ABI 7500—Applied Biosystem) and a miScript miRNA PCR Primers (Qiagen). The miRNAs’ expression was normalized to the expression of Hs_RNU6-2.

### Data analysis

All in vitro experiments were performed at least three times (*n* = *3*). Statistical analysis was performed using GraphPad InStat version 3.0a for Macintosh (GraphPad Software, San Diego, CA, USA). ANOVA was used to estimate the effects of chemicals, time, and doses and to compare the between-group differences. Dunnett’s comparison test was used for multiple comparisons after analysis of variance. Student’s t test was used for human samples. Kolmogorov–Smirnov and Shapiro–Wilk tests were used as normality tests. Differences were considered significant at *p* ≤ 0.05.

## Results

### miRNA profile and expression induced by contact allergens on iDCs

**Fig. 1:**
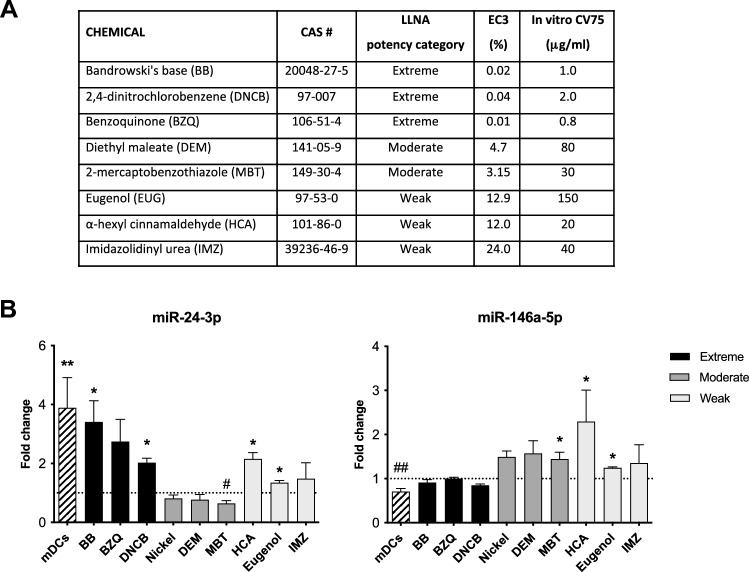
miR-24-3p and miR-146a-5p expression after iDCs exposure to contact allergens of different potency. **A** List of chemicals tested and CV75 obtained on THP-1 after 24 h of exposure. CV75 represents the concentration resulting in 75% cell viability as assessed by propidium iodine staining and FACS analysis. **B** iDCs were exposed for 24 h to selected contact allergens using the CV75 as final concentration and to the maturation cocktail (rhIL-4 3000 UI/mL, rhGM-CSF 1500 UI/mL, rhTNF-α 2000 UI/mL and ionomycin 200 ng/mL). Results about miRNAs expression are expressed as fold change (2^−ΔΔ*Ct*^). Each value represents the mean of *n* = *3* independent experiments. Statistical analysis was performed with a Dunnett’s comparison test, with *^,#^*p* < 0.05 and **^,##^*p* < 0.01 vs control vehicle-treated cells (* was used for statistically significant increase expression; ^#^ was used for statistically significant decrease expression). Dotted line is set up at 1.0 (control)

The Script miRNA PCR Array Human miFinder was initially performed profiling the 84 miRNAs. After miFinder Array analysis, data were insert in a miScript miRNA PCR Array Data Analysis excel file (provided by Qiagen). After a bibliographic research made to investigate miRNAs possibly involved in allergic contact dermatitis and/or immune system activation/de-regulation, six miRNAs were selected for the further analysis. In details: hsa-miR-24-3p, 27a-3p, 140-3p, 146a-5p, 223-3p, and let-7g-5p. The miRNAs’ expression was normalized to the expression of Hs_RNU6-2. Results reported in Table 1 show that the extreme contact allergen BB significantly up-regulated the expression of miR-24-3p, and down-regulated miR-140-3p and miR-146a-5p. While the weak contact allergen HCA induced a statistical significant increase in both miR-140-3p and miR-146-5p expression, and was able to down-regulate miR-27a-3p expression. The effect induced by the extreme contact allergen BB was overall similar to the one obtained in iDCs exposed to the maturation cocktail (mDCs). Based on the different expression induced by the two contact allergens of different potency and also supported by literature evidence related to ACD, two miRNAs have been then selected to proceed with further analysis: miR-24-3p and miR-146a-5p. Additional skin sensitizers were tested using the concentration resulting in 75% cell viability (CV75) (Fig. 1, Panel A), previously assessed by propidium iodide staining and cytometer analysis. In total, nine contact sensitizers were used, three for each potency class as reported in Fig. 1, Panel A. Results reported in Fig. 1, Panel B show a consistent similar behavior among the different potency classes. In fact, miR-24-3p expression was induced by the extreme and interestingly also by the weak contact allergens, while moderate contact allergens down-regulate this miRNA expression (MBT in statistical significant manner). On the contrary, miR-146a-5p was down-regulated only by extreme contact sensitizers.

**Table 1:** miRNAs expression after iDCs exposure to BB (extreme), HCA (weak) and to the maturation cocktail (mDCs)

	BB^1^	HCA^2^	mDCs^3^
miR-24-3p	3.41 ± 1.24*	2.15 ± 0.38	3.88 ± 1.77*
miR-27a-3p	0.90 ± 0.11	0.46 ± 0.08^##^	1.70 ± 0.55*
miR-140-3p	0.97 ± 0.05	2.35 ± 0.54***	0.86 ± 0.06
miR-146a-5p	0.91 ± 0.12	2.29 ± 1.24**	0.71 ± 0.12
miR-223-3p	1.20 ± 0.37	1.46 ± 0.50	1.26 ± 0.10^#^
miR-let-7g-5p	1.04 ± 0.36	0.93 ± 0.14	0.44 ± 0.08^#^

iDCs cells were exposed for 24 h to the extreme contact allergen BB 1 μg/mL, to the weak contact allergen HCA 20 μg/mL and to the maturation cocktail (rhIL-4 3000 UI/mL, rhGM-CSF 1500 UI/mL, rhTNF-α 2000 UI/mL and ionomycin 200 ng/mL). Results are expressed as fold change (2^−ΔΔ*Ct*^). Each value represents the mean of *n* = 3 independent experiments. Statistical analysis was performed with a Dunnett’s comparison test, with *^,#^*p* < 0.05, **^,##^*p* < 0.01 and ***p < 0.001 vs control vehicle-treated cells (* was used for statistically significant increase expression; ^#^ was used for statistically significant decrease expression)

^1^Bandrowski’s base

^2^α-Hexylcinnamaldehyde

^3^Mature dendritic cells

### *Role of miR-24-3p and miR-146a-5p in the *in vitro* iDC maturation process*

**Fig. 2:**
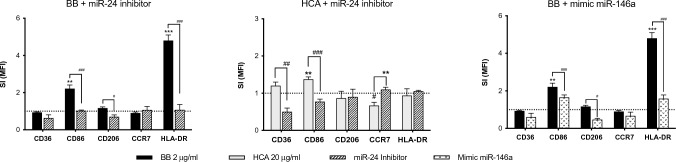
Cell surface markers expression after iDCs pre-treatment with miR-24-3p inhibitor and mimic-miR-146a-5p. iDCs were exposed for 24 h to BB (2 μg/mL) and HCA (20 μg/mL) in presence or absence of the miR-24-3p inhibitor (50 nM) or miRNA mimic (5 nM). After 24, the expression of CD36, CD86, CD206, CCR7 and HLA-DR were evaluated. Results are expressed as stimulation index (treatment/control). Each value represents the mean of *n* = 3 independent experiments. Statistical analysis was performed with a Dunnett’s comparison test, with *^,#^*p* < 0.05, **^,##^*p* < 0.01 and ***^, ###^*p* < 0.001 vs control cells (* was used for statistically significant increase expression; ^#^ was used for statistically significant decrease expression). Dotted line is set up at 1.0 (control)

To support the effective involvement of miR-24-3p and miR-146a-5p in the process of iDCs maturation in the proposed in vitro assay, specific inhibitor/mimic miRNAs were used. Firstly, CD36, CD86, CD206, HLA-DR and CCR7 expression were evaluated to support the effective maturation process. Then, the possible involvement of the selected miRNAs in the cell surface marker expression was evaluated. To investigate the functional role of miR-24 up-regulated by the extreme contact allergen BB and by the weak sensitizer HCA, a specific suppressor of this miRNA has been used. On the contrary, the mimic miR-146a, designed to mimic the endogenous miRNA, was used due to the down-regulation induced by BB. In Fig. 2, cell surface markers expression after 24 h are reported. The presence of the miR-24 inhibitor lead to a statistical significant reduction of CD86 and HLA-DR in cells exposed to BB suggesting an involvement of miR-24 in the expression of these cell surface markers. About the weak contact sensitizer HCA, miR-24 inhibitor was able to reduce in statistical significant manner the expression of CD36 and CD86. Also for miR-146a expression, the same cell surface markers high lightened with the miR-24 inhibitor were modulated (i.e. CD86, CD206 and HLA-DR) by BB exposure.

Overall data collected suggest that both miRNAs seems to be involved in the maturation process of DC in our in vitro system. Anyway, differences between the two contact allergens of different potency emerged. The expression (and consecutively the modulation) of some markers is different between the two contact allergens (i.e. CD36 and HLA-DR), considering also the intensity of the induction.

### Role of PKCβ in miR-24-3p and miR-146a-5p expression

**Fig. 3:**
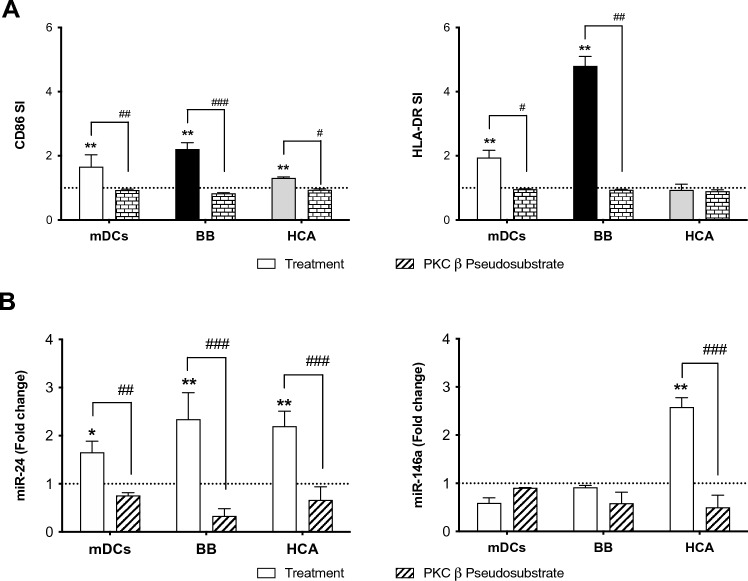
Role of PKCβ in chemical allergen-induced miR-24-3p and miR-146a-5p expression. iDCs were pre-treated for 2 h with a specific PKCβ inhibitor (PKCβ pseudosubstrate—5 μM final concentration) and then were exposed for 24 h to BB (1 μg/mL), HCA (20 μg/mL) and to the maturation cocktail (rhIL-4 3000 UI/mL, rhGM-CSF 1500 UI/mL, rhTNF-α 2000 UI/mL and ionomycin 200 ng/mL). Results are expressed as fold change (2^−ΔΔ*Ct*^). Each value represents the mean of *n* = 3 independent experiments. Statistical analysis was performed with a Dunnett’s comparison test, with *p < 0.05, **^,##^*p* < 0.01 and ^###^*p* < 0.001 vs control cells (* was used for statistically significant increase expression; ^#^ was used for statistically significant decrease expression). Dotted line is set up at 1.0 (control)

In particular, data reported in Fig. 3 showed a complete down-regulation of miR-24-3p after PKCβ inhibition. The inhibition of PKCβ lead to a reversion of miR-146a-5p expression after HCA treatment (down-regulation), while in BB cells’ treatment conditions, the down-regulation of the miRNA with the contact sensitizer alone was confirmed but not reverted by the use of PKCβ substrate. Results obtained support the important role of PKCβ in DCs maturation process in the proposed in vitro model, giving additional information about miR-24-3p and miR-146a-5p involvement.

### Role of miR-24-3p and miR-146a-5p in human samples

**Fig. 4:**
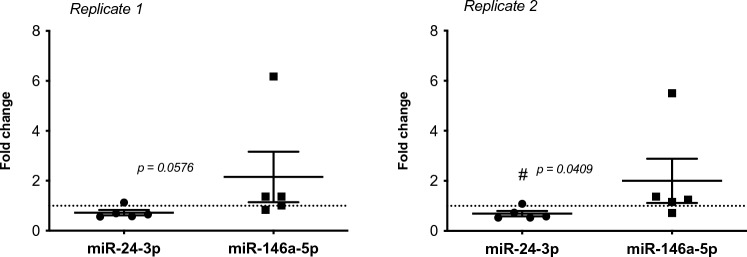
*In human* correlation of miR-24-3p and miR-146a-5p expression. Human samples derived from 5 patients patch tested with nickel for 72 h were used to analyze the expression of miR-24-3p and miR-146a-5p. Results are expressed as fold change (2^−ΔΔ*Ct*^) and the two technical replicates are reported. Each value represents the five different patients (*n* = *5*). Statistical analysis was performed with Student’s *t* test, with ^#^*p* < 0.05 control. Dotted line is set up at 1.0 (control)

The expression of the miR-24 and miR-146a, previously analyzed in vitro, was performed also in RNA samples obtained from human patch test to nickel. In Fig. 4, human data have been reported. In our human data, miR-24 resulted down-regulated by Nickel, suggesting a pro-inflammatory role played by this miRNA and in line with what has been obtained by in vitro experiments.

## Discussion

The main aim of this work was to identify and investigate the role of miRNAs, and to better understand the mechanism underlying the DCs maturation process in the field of the ACD. The attention was focused on two miRNAs, namely miR-24-3p and miR-146a-5p, and on the investigation of their involvement in the activation of DCs after exposure to contact allergens of different potency.

DCs, acting as sentinel, migrate as immature precursor cells into secondary lymphoid organs and undergo a complex maturation process induced by signals generated by local inflammation or microbial infection. When maturation-inducing stimuli are sensed by DCs, MHC class II and co-stimulatory molecules cell surface expression increase, initiating the antigen-specific T-cell responses (Banchereau and Steinman 1998). Different approaches based on the use of DC like cultures have been developed to mimic the maturation process of DCs. Among them, the THP-1 cell line is one of the most interesting alternatives because of its availability and abundance (Yoshida et al. 2003). It has also been demonstrated using diverse combinations of cytokines and hematopoietic differentiation agents, that THP-1 cells can be easily differentiated into immature and mature DCs with the phenotypic, molecular, and functional properties of primary DCs generated from human donor-derived CD14 + monocytes or CD34 + HPCs (Berges et al. 2005). This model has been used in the current study starting from previous evidences showing that the only the exposure to extreme allergens was sufficient to provide the three signals required for T-cell activation. Moderate and weak allergens while inducing Signal 2 and in some instances, also Signal 3, failed to induced HLA-DR (Galbiati et al. 2020).

MiRNAs are central regulators of post-transcriptional gene expression and play important role in many pathologies and it has been demonstrated their involvement also in a variety of skin diseases, including ACD (Løvendorf and Skov 2015; Gulati et al. 2015). Mechanistic studies of miRNAs targeting and function are only beginning to emerge and it is unlikely that one miRNA alone holds the key to explain the pathology of ACD (Mehta and Baltimore 2016; Bartel 2018). Different miRNAs have been increasingly recognized as important modulators of Th cells (Th1, Th2, Th17, and Tfh) and Tregs differentiation (Naqvi et al. 2015). In particular, miR-24-3p is involved in the inhibition of IL-4 production in T cells (Baumjohann and Ansel 2013; Fayyad-Kazan et al. 2014). Recent evidence reports that *TRAF6* is a direct target of miR-24-3p in LPS-induced inflammatory responses with attenuation of NF-κB/MAPK signaling pathway (Oladejo et al. 2022). What in general emerged from a literature research, not focus only on ACD and immune system, but also in other pathologies, is that the role played by miR-24-3p is a pro-inflammatory one. In fact, in several paper, it has been reported that the over expression of miR-24-3p lead to an anti-inflammatory status or an amelioration of several parameters such as cell viability, injury status, specific enzyme activation, and in general inflammation process (Fayyad-Kazan et al. 2014; Xu et al. 2021; Jiang et al. 2021). Results obtained in our experiments with nickel, both in in vitro assay and *in human* samples, support the literature evidences. Nickel was able to induce a down-regulation of miR-24, inducing an increase in the expression of several cell surface markers, such as CD36, CD86, CCR7 and HLA-DR clearly involved in the DC maturation process. What remains unclear and unexpected is the behavior of the extreme and weak contact allergens that were able to induce the up-regulation of miR-24. One possible speculation could be related to the time point evaluated with different considerations related to the potency of the sensitizers tested.

Literature evidence reported that miR-146-5p is involved in TLR4-MyD88 signaling regulations pathway (Curtale et al. 2010). The inhibition of TLR4, which is important in ACD and DC activation by allergens, may indicate a reduced functional maturation. In particular, both miR-146a and miR-146b have been shown to target TRAF6, IRAK1 and IRAK2, all of which are components of the so-called Myddosome, a stoichiometric defined protein scaffold involved in the regulation of the cross-talk observed in the TLR signaling pathways (Hou et al. 2009; Motshwene et al. 2009). It has been shown that miR-146a expression is directly controlled by NF-κB binding to its promoter (Taganov et al. 2006) and further studies indicate that this miRNA may also target FADD, another critical regulator of T cells (Motshwene et al. 2009; Nejad et al. 2018; Rusca and Monticelli 2011). The effective involvement of miR-146a-5p in the iDCs maturation process induced by contact allergen was confirmed in the proposed in vitro study, demonstrating that several crucial cell surface markers involved in DC maturation were modulated by the use of the specific mimic miRNA. Potency refers to the intrinsic property of a sensitizing chemical and is based on the concentration of chemical needed to induce a positive response (Kimber and Pemberton 2014). Maturation of DCs in response to sensitizing agents has been identified as one of the in vitro strategies to predict the sensitizing capacity of chemicals (Wong et al. 2015). Results obtained in this study shown a consistent similar behavior among the different potency classes and the two miRNAs evaluated. In particular, miR-146a resulted differently modulated by contact allergens of different potency resulting down-regulated only by the extreme contact allergens and up-regulated by moderate and weak ones. Furthermore, iDCs exposed to extreme contact allergens were able to induce a modulation of miRNAs similar to what observed in mature dendritic cells (mDCs) supporting our previous observation about a different kinetic in DCs maturation based on the hypothesis that extreme contact allergens are able to lead to a full maturation of iDCs.

Another important aim of this study was to investigate the possible involvement of PKCβ in miR-24-3p and miR-146a-5p expression induced by contact sensitizers. We previously demonstrated the role of RACK-1 and PKCβ in chemical allergen-induced CD86 expression and IL-8 release in THP-1 cells and in primary human dendritic cells. Three contact sensitizers were selected and a selective cell-permeable inhibitor of PKCβ and the broad PKC inhibitor GF109203X completely prevented chemical allergen-induced CD86 expression and significantly modulated IL-8 release (Corsini et al. 2014). The ability of the PKCβ pseudosubstrate to completely prevent miR-24-3p and miR-146a-5p (this one only for weak allergen tested) expression confirms the role of PKCβ activation in the initiation of chemical allergen-induced DC activation and maturation.

Nickel is one of the most common allergen and the nickel allergy is recognized as a type of ACD (Rishor-Olney et al. 2022). A recent integrative analysis of miRNA/mRNA expression profile in human skin patch test report a clear different miRNA signature induced by allergens compare to irritants and results obtained show an up-regulation of miR-155-5p in patch test samples induced by nickel sulfate exposure (Werner et al. 2021 Apr). In this paper, the presence of the nickel in both conditions, in vitro and *in human*, creates a useful bridge to connect the generated data. Even if not statistically significant an increased expression of miR-146a in human samples expression could be appreciated and a down-regulation of miR-24 is reported.

The proposed in vitro model demonstrates that the up-regulation of miR-24-3p and the down-regulation of miR-146a-5p lead to the activation and maturation of the DCs induced by contact sensitizers through the involvement of the PKCβ.

## Data Availability

Not applicable.
